# Evaluation of single-view contrast-enhanced mammography as novel reading strategy: a non-inferiority feasibility study

**DOI:** 10.1007/s00330-019-06215-7

**Published:** 2019-05-09

**Authors:** M. B. I. Lobbes, J. Hecker, I. P. L. Houben, R. Pluymakers, C. Jeukens, U. C. Laji, S. Gommers, J. E. Wildberger, P. J. Nelemans

**Affiliations:** 1 Department of Radiology and Nuclear Medicine, Maastricht University Medical Center, P.O. Box 5800, 6202 AZ Maastricht, The Netherlands; 2 GROW School for Oncology and Developmental Biology, Maastricht University, Maastricht, The Netherlands; 3 Department of Epidemiology, Maastricht University, Maastricht, The Netherlands

**Keywords:** Breast cancer, Screening, Mammography

## Abstract

**Background:**

Guidelines recommend screening of high-risk women using breast magnetic resonance imaging (MRI). Contrast-enhanced mammography (CEM) has matured, providing excellent diagnostic accuracy. To lower total radiation dose, evaluation of single-view (1 V) CEM exams might be considered instead of double-view (2 V) readings as an alternative reading strategy in women who cannot undergo MRI.

**Methods:**

This retrospective non-inferiority feasibility study evaluates whether the use of 1 V results in an acceptable sensitivity for detecting breast cancer (non-inferiority margin, − 10%). CEM images from May 2013 to December 2017 were included. 1 V readings were performed by consensus opinion of three radiologists, followed by 2 V readings being performed after 6 weeks. Cases were considered “malignant” if the final BI-RADS score was ≥ 4, enabling calculation of sensitivity, specificity, and area under the receiver operating characteristic curve (AUC). Histopathological results or follow-up served as a gold standard.

**Results:**

A total of 368 cases were evaluated. Mean follow-up for benign or negative cases was 20.9 months. Sensitivity decreased by 9.6% from 92.9 to 83.3% when only 1 V was used for evaluation (*p* < 0.001). The lower limit of the 90% confidence interval around the difference in sensitivity between 1 V and 2 V readings was − 15% and lies below the predefined non-inferiority margin of − 10%. Hence, non-inferiority of 1 V to 2 V reading cannot be concluded. AUC for 1 V was significantly lower, 0.861 versus 0.899 for 2 V (*p* = 0.0174).

**Conclusion:**

Non-inferiority of 1 V evaluations as an alternative reading strategy to standard 2 V evaluations could not be concluded. 1 V evaluations had lower diagnostic performance compared with 2 V evaluations.

**Key Points:**

*• To lower radiation exposure used in contrast-enhanced mammography, we studied a hypothetical alternative strategy: single-view readings (1 V)* versus *(standard) double-view readings (2 V).*

*• Based on our predefined margin of − 10%, non-inferiority of 1 V could not be concluded.*

*• 1 V evaluation is not recommended as an alternative reading strategy to lower CEM-related radiation exposure.*

## Introduction

Breast cancer is a leading cause of cancer-related deaths in women worldwide every year. Some women have genetic mutations, making them more susceptible to develop breast cancer in their life. These include, for example, *BRCA-1*, *BRCA-2*, *TP53*, *PALB2*, *CDH1*, *STK11*, and *PTEN* gene mutations. Other reasons for having a > 20% lifetime risk of developing breast cancer include prior chest (mantle) radiation and specific syndromes, such as Li Fraumeni or Cowden syndrome. Based on studies that showed an improved cancer detection rate in these women when breast MRI is used as adjunct screening modality [1–3], current international guidelines recommend annual screening of these women with breast MRI [4–6].

The use of breast MRI as a screening tool has some limitations. It is a relatively expensive modality and a widespread use as a screening tool is challenging due to the limited availability of sufficient scan slots. Breast MRI has an excellent sensitivity, but its specificity is moderate (resulting in false-positive findings requiring additional follow-up exams or biopsies) [7]. In addition, studies have shown that gadolinium (Gd) of contrast agents accumulates in the body [8]. Although no negative long-term side-effects have been reported, this phenomenon might result in a discouragement of using Gd-based contrast agents for (repeated) screening purposes. Finally, a number of women will not be able to undergo breast MRI because of claustrophobia, previous adverse reactions to the contrast agent used, or the presence of metal objects within their bodies. Therefore, an alternative imaging modality might be appealing for these groups of women, using for example contrast-enhanced mammography (CEM, synonyms: CESM, contrast-enhanced spectral mammography or CEDM, contrast-enhanced dual-energy mammography).

The underlying principle of CEM is comparable to that of breast MRI: growing tumors need to sprout newly formed blood vessels to adhere to their increasing demand for nutrients in a process called angiogenesis [9]. These newly formed vessels are rapidly formed and “leaky” to contrast agents like the ones used in CEM or breast MRI [10]. These contrast agents can extravasate into the tumor interstitium, causing enhancement on CEM or MRI exams. Multiple studies have evaluated the diagnostic performance of CEM compared with breast MRI, showing that sensitivity is at least equal to breast MRI [11–13]. CEM is increasingly considered as a potential screening modality, especially in women with high or intermediate breast cancer risk or dense breasts [14, 15]. However, disadvantages of CEM over breast MRI, especially when it would be considered in screening of patients, are not only the use of iodinated contrast agents but also its increased radiation dose (for example, when compared with full-field digital mammography or FFDM) [16] and the lack of CEM-guided biopsy capabilities.

To compensate for the increased radiation dose in screening patients at high risk for developing breast cancer using CEM, we propose an alternative strategy: single-view CEM. In this retrospective study, we evaluated the diagnostic performance of single-view CEM (1 V) versus standard double-view CEM (2 V) to find out if this approach has the potential to serve as an alternative strategy. This retrospective study was designed as a non-inferiority study to evaluate whether use of 1 V results in an acceptable sensitivity for detecting breast cancer in our study population, while maintaining similar specificity when compared with 2 V. However, we also consider this to be a feasibility study, as our primary analyses were not conducted on the assumed target population of women at high risk of developing breast cancer, but on our institute’s available CEM database.

## Materials and methods

For this study, we retrospectively analyzed all CEM exams performed at our institute between May 2013 and December 2017. Indications for CEM included recalls after a positive screening mammography, suspicious findings during physical examination or detected on imaging performed elsewhere, unknown primary tumors, and inconclusive findings at FFDM or alternative to breast MRI. All images were anonymized with an allocated study code. Due to the study design used, the necessity to acquire informed consent was waived by our ethical committee (decision number METC 15-4-008).

### Image protocol and gold standard

All CEM exams were performed on a single CEM unit (Senographe* Essential with Senobright* upgrade, GE Healthcare) using a non-ionic, monomeric, low-osmolar contrast agent at a dose of 1.5 ml/kg of body weight (iopromide, Ultravist 300, Bayer Healthcare). Iodinated contrast was administered intravenously with a flow rate of 3 ml/s 2 min prior to image acquisition. The breasts were imaged in (at least) mediolateral oblique and craniocaudal views.

For all solid lesions and (micro)calcifications, histopathological results served as the gold standard. In cases of negative results or suspected cysts, a minimum follow-up of 12 months was used to exclude any false-negative findings.

### Image analysis

The CEM images were evaluated using a double-reading strategy, which is the applied strategy for our nationwide screening program. In short, two certified screening radiologists (with 9 and 7 years of screening expertise and both having 5 years of CEM experience) provided a BI-RADS classification for the 1 V images first (i.e., mediolateral oblique view for both breasts, as this view covers the largest part of the breast). For this study, we considered a BI-RADS classification of ≥ 4 to be “suspicious for breast cancer,” which in a screening setting would require a recall. BI-RADS classifications ≤ 3 were considered “not suspicious for breast cancer” and would not have been recalled in a screening setting. For the primary analysis, a consensus opinion was used and in case of discrepancies between both readers, a third screening-certified reader (6 years of screening expertise and 5 years of CEM experience) was consulted for the final decision. To minimize recall bias, all cases were re-evaluated in a similar fashion after 6 weeks, but this time, the complete CEM exam was available (2 V). The images were evaluated in a different, randomized order in these two sessions. During the evaluations, all radiologists were blinded to the primary CEM indication, their score in the other reading session and final diagnosis.

### Statistical analysis

The study was designed as a non-inferiority study to evaluate whether the use of single-view CEM exams does not result in an unacceptable lower sensitivity for detecting breast cancer in our study population, while maintaining similar specificity when compared with double-view CEM exams. The pre-specified non-inferiority margin was determined at 10%. This margin was chosen because a sensitivity decrease by more than 10% was considered unacceptable.

Assuming a sensitivity in the reference group (i.e., 2 V group) for the detection of malignant cases of 90%, 112 pairs with malignant cases are required to be 80% sure that a one-sided 95% confidence interval (CI, equivalent to a two-sided 90% CI) will exclude a difference in favor of the 2 V group of more than 10%. Prior to this study, we estimated the prevalence of malignant cases to be 30% [17], resulting in a required total sample size of 373 cases (source: OpenEpi, www.openepi.com).

As explained before, BI-RADS 1–3 were considered benign and BI-RADS 4–5 malignant. Using these cutoff values, sensitivity, specificity, positive predictive value (PPV), and negative predictive value (NPV) were calculated. In addition, the area under the ROC curve (AUC) was calculated for the different reading sessions.

The absolute differences in sensitivity and specificity and one-sided 95% CI (equivalent to two-sided 90% CI) of the difference were calculated using Tango’s score CI for a difference of paired proportions [18]. The corresponding one-sided *p* values were derived using McNemar’s test for paired proportions. The paired areas under the curve (AUC) of the receiver operating characteristic (ROC) curves for 1 V and 2 V view exams were compared using an algorithm developed by DeLong et al [19].

STATA (version 13.1, StataCorp LLC) and R (version 2.15.1, The R Foundation for Statistical Computing) were used for the statistical analyses. One-sided *p* values of 5% were considered to indicate statistical significance.

## Results

The mean age of the women included in our study population was 59.7 years (range 50–77 years). In the study period, 368 patients instead of the required 373 patients were included, but the prevalence of malignancies turned out to be higher than expected (34.2%). Consequently, the number of malignant cases was 126, which is higher than the planned sample size of 112 cases for the evaluation of the sensitivity difference. Of the 126 malignant diagnoses, 48 consisted of invasive cancer of no special type (NST, or invasive ductal carcinoma; 13%), followed by 42 cases of ductal carcinoma in situ (DCIS, 11.4%) and 30 cases of invasive lobular carcinoma (ILC, 8.1%). The remaining 6 cases were invasive breast cancers not otherwise specified (1.7%). Of the benign diagnoses, most were cysts (*n* = 90), followed by fibroadenoma (*n* = 19) and intramammary lymph nodes (*n* = 10), with the remaining benign diagnosis being not otherwise specified (*n* = 35). A total of 88 cases were negative. The mean follow-up period for benign or negative cases was 20.9 months, range 12.7–55.3 months.

**Fig. 1:**
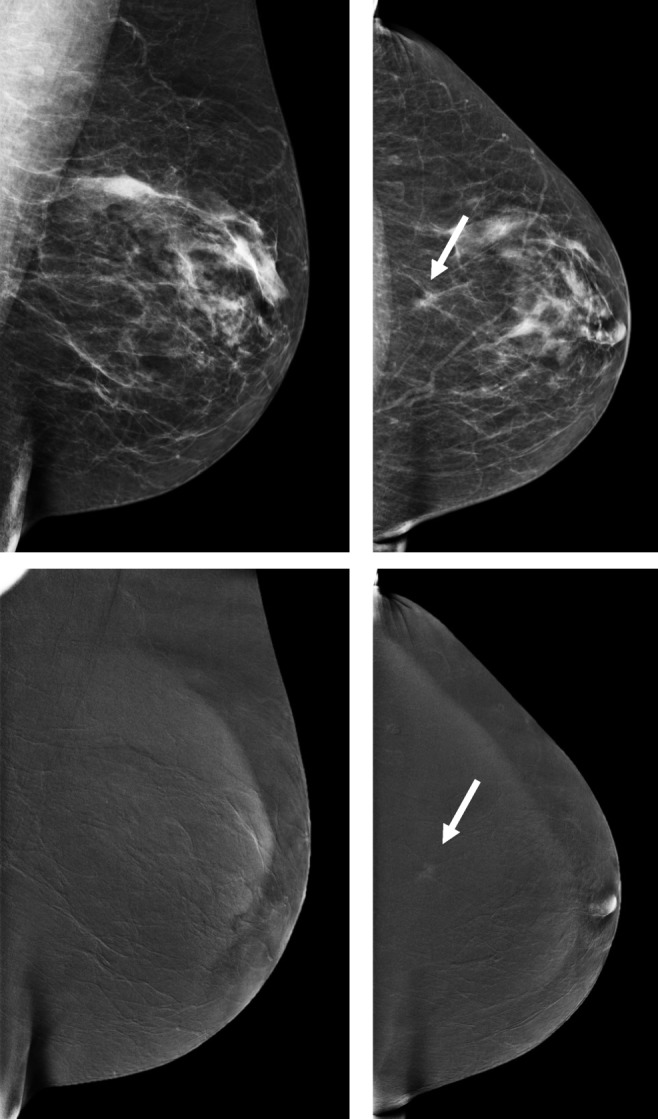
Example of an invasive lobular cancer in the left breast, not detected in 1 V (MLO), only detected on 2 V as an ill-defined focal asymmetry that showed slight enhancement on the recombined images (arrow)

The results for the 1 V and the 2 V evaluation are presented in Table 1. For the 1 V readings, there were 48 discrepancies between the first two readers, while the number of discrepancies for the 2 V readings was 64. The combined assessment of the radiologists based on 1 V CEM images resulted in 105 true-positive cases (TP), 38 false-positive cases (FP), 204 true-negative cases (TN), and 21 false-negative cases (FN). For the 2 V evaluation, these numbers were the following: 117 TPs, 50 FPs, 192 TNs, and 9 FNs, respectively. An overview of the histopathological results of the FN cases is presented in Table 2. An example of an invasive lobular carcinoma that was overlooked on the 1 V (MLO) exam and detected on 2 V readings is presented in Fig. 1.

**Table 1:** Comparison of sensitivity and specificity between double-view (2 V) and single-view (1 V) contrast-enhanced mammography exams. Results are presented for the combination of all three readers (R1 + R2 + R3) and for the first (R1) and second (R2) reader independently

	Double (2 V) view % (*n*)	Single (1 V) view % (*n*)	Difference % 1 V minus 2 V (90% CI)	*p* value (one-sided)
Sensitivity				
R1 + R2 + R3	92.9 (117/126)	83.3 (105/126)	− 9.6 (− 15 to − 5.3)	< 0.001
R1	92.1 (116/126)	76.2 (96/126)	− 15.9 (− 22 to − 10)	< 0.001
R2	92.9 (117/126)	82.5 (104/126)	− 10.4 (− 16 to − 5)	0.0012
Specificity				
R1 + R2 + R3	79.3 (192/242)	84.3 (204/242)	+ 5.0 (1.2 to 8.9)	0.0251
R1	65.7 (159/242)	84.3 (204/242)	+ 18.6 (14 to 23)	< 0.001
R2	72.7 (176/242)	76.9 (186/242)	+ 4.2 (− 0.1 to 8.5)	0.07165

*CI*, confidence interval

**Table 2:** Overview of histopathological diagnoses of false-negative cases

Missed on view	Lesion type	Tumor type	Size (mm)	Grade	ER	PR	HER2
1 V	Calcifications	DCIS	8	2	N/A	N/A	N/A
1 V	Calcifications	DCIS	12	2	N/A	N/A	N/A
1 V	Calcifications	DCIS	10	1	N/A	N/A	N/A
1 V	Calcifications	DCIS	25	2	N/A	N/A	N/A
1 V	Calcifications	DCIS	14	3	N/A	N/A	N/A
1 V	Calcifications	DCIS	30	1	N/A	N/A	N/A
1 V	Calcifications	DCIS	5	3	N/A	N/A	N/A
1 V	Mass	ILC	13	2	Positive	Positive	Negative
1 V	Mass	ILC	6	2	Positive	Positive	Negative
1 V	Mass	ILC	5	2	Positive	Positive	Negative
1 V	Mass	NST	8	2	Positive	Positive	Negative
1 V	Mass	Papillary	4	1	Positive	Positive	Negative
2 V	Calcifications	DCIS	20	2	N/A	N/A	N/A
Both	Calcifications	DCIS	10	3	N/A	N/A	N/A
Both	Calcifications	DCIS	15	3	N/A	N/A	N/A
Both	Calcifications	DCIS	48	3	N/A	N/A	N/A
Both	Calcifications	DCIS	8	2	N/A	N/A	N/A
Both	Calcifications	DCIS	12	2	N/A	N/A	N/A
Both	Calcifications	DCIS	2	2	N/A	N/A	N/A
Both	Mass	ILC	15	2	Positive	Positive	Negative
Both	Mass	NST	8	1	Positive	Negative	Negative

*ER*, estrogen receptor; *PR*, progesterone receptor; *HER2*, human epidermal growth factor-2; *DCIS*, ductal carcinoma in situ; *ILC*, invasive lobular carcinoma; *NST*, invasive carcinoma of no special type

**Fig. 2:**
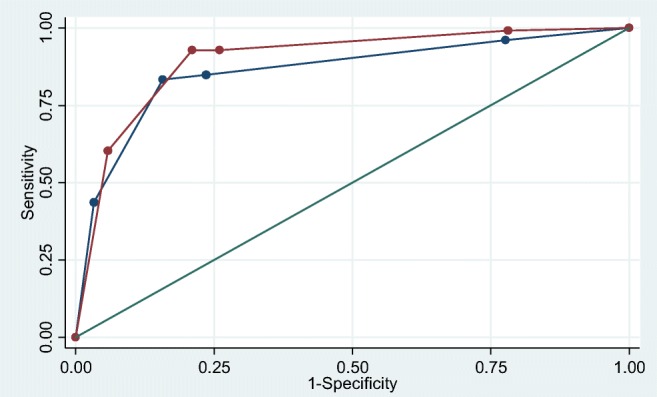
Receiver operating characteristic (ROC) curves for the consensus results (R1 + R2 + R3) based on the 1 V reading strategy (blue line) and the 2 V reading strategy (red line). The area under the ROC curve (AUC) for 1 V readings was significantly lower (0.861) than the (standard) 2 V readings (0.899, one-sided *p* value 0.0174)

Our results show that sensitivity decreases when only 1 V is used for the evaluation of the CEM images. This decrease is statistically significant (*p* < 0.001). The lower limit of the 90% CI of the difference in sensitivity between 1 V and 2 V readings is − 15% and lies below − 10%, but the entire 90% CI (ranging from − 15 to − 5.3%) does not exclude the non-inferiority margin. More specifically, non-inferiority could be observed for the first reader, but could not be concluded for the second or the combined readings. It can also be observed that there is a trade-off between sensitivity and specificity: the significant decrease of sensitivity is accompanied by a significant increase in specificity. These trends are observed for the evaluations by R1 and R2 as well. To evaluate whether overall diagnostic performance decreases when using the 1 V strategy instead of the standard 2 V evaluation, AUCs were compared. The ROC curves for the consensus results (R1 + R2 + R3 based on 1 V and 2 V readings) are presented in Fig. 2. The AUC for 1 V was 0.861 versus 0.899 for 2 V (one-sided *p* = 0.0174), indicating a significantly worse overall diagnostic performance for the 1 V readings when compared with 2 V.

These results, however, were based on the analyses performed on our institute’s CEM database, which is not the assumed target population of women at high risk for developing breast cancer (see also the “Study limitations” section).

## Discussion

In this study, the sensitivity of 1 V evaluations was significantly lower compared with (standard) 2 V evaluations. The lower CI limit of the difference in sensitivity between 1 V and 2 V lies below the predefined non-inferiority margin of − 10% and, consequently, the results did not allow for the conclusion that 1 V evaluation is non-inferior to 2 V evaluation of CEM exams. We observed a trade-off between sensitivity and specificity: the significant decrease of sensitivity was accompanied by a significant increase in specificity. These trends were also observed for the evaluations by R1 and R2. Using 1 V readings instead of 2 V readings leads to a substantial and significant decrease of sensitivity and overall diagnostic performance. The majority of FN diagnoses were caused by DCIS and ILC (Table 2). Based on our observations, we would not recommend 1 V evaluations as an alternative reading strategy to lower CEM-related radiation exposure.

Since previous studies have demonstrated an improved cancer detection rate in women at high risk for developing breast cancer who were annually screened with breast MRI, many guidelines have recommended its use for this indication. In our national guidelines, annual breast MRI screening is recommended for most women with a known genetic predisposition for developing breast cancer, such as *BRCA-1*, *BRCA-2*, *TP53*, *PALB2*, *CDH1*, *STK11*, and *PTEN* gene mutations. Other women eligible for this kind of screening are those with prior chest (mantle) radiation or with Li Fraumeni or Cowden syndrome. Screening is initiated at the age of 25 or 30 (depending on the gene mutation) and continues to the age of 60 [20]. Consequently, all these women undergo approximately 30–35 screening breast MRI exams in their lives.

Dynamic, gadolinium (Gd)-enhanced T1w images are the backbone of a breast MRI protocol. McDonald et al observed an accumulation of Gd within the brain, even in patients with normal renal function and without any intracranial abnormalities [21, 22]. Although confirmed by other studies, there is no evidence at present to show any adverse effects [8], but several international bodies have recommended a more cautious use of these agents until long-term effects can be ruled out.

**Fig. 3:**
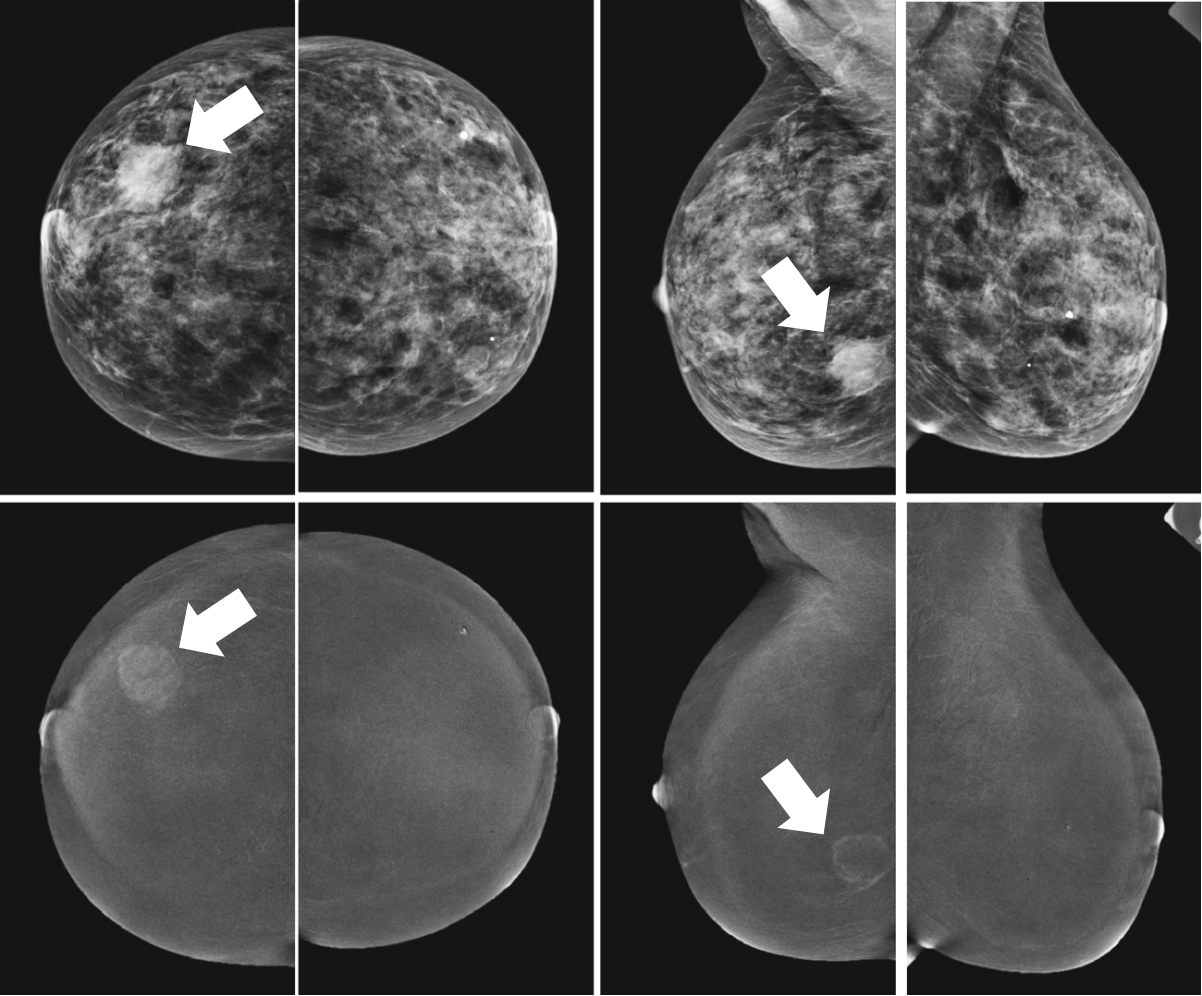
Typical example of a contrast-enhanced mammography exam, showing the low-energy images in the top row and the recombined (contrast-enhanced) images on the bottom row. In this case, an irregular, ill-defined mass is visible in the outer lower quadrant of the right breast (arrow), showing rim enhancement after contrast administration. Biopsy revealed an invasive carcinoma of no special type (NST)

CEM might be considered in the future as an alternative screening modality for this group of women, for example for those who have claustrophobia, refuse the repeated administration of Gd-based contrast agents, or who prefer CEM over breast MRI. In CEM, an iodine-based contrast agent is administered intravenously 2 min prior to image acquisition. By using a dual-energy technique, the radiologist can read a low-energy image (which is like a conventional full-field digital mammogram) and a recombined image, in which areas of enhancement can be appreciated (Fig. 3) [23]. Previous studies have shown that CEM is consistently superior to FFDM, with comparable diagnostic performance to breast MRI in terms of both cancer detection and the evaluation of disease extent [11–13, 24].

Jochelson et al were the first to evaluate the potential of CEM as a screening tool for high-risk patients in a study containing 307 cases [14]. In the first screening round, three cancers (two invasive and one DCIS) were detected. Breast MRI detected all three, whereas CEM detected only the two invasive breast cancers. None of the cancers were visible on the low-energy images. After the next screening round (after 2 years), five additional screen-detected cancers were observed. The PPV turned out to be comparable between CEM and breast MRI: 15% and 14%, respectively. Hence, the authors concluded that CEM might be a suitable alternative for screening these women when they had a contra-indication for breast MRI or who have limited access to it. However, an important limitation of using CEM as a screening tool over MRI is the current lack of (commercially available) CEM-guided stereotactic capabilities. This is expected to change soon, as prototypes are currently being evaluated for clinical applications. Nevertheless, they are not available at this point, which further supports the fact that at this point, screening of high-risk women using CEM can only be recommended when breast MRI is contra-indicated.

More recently, Sorin et al studied the diagnostic accuracy of CEM compared with FFDM in women with dense breasts and intermediate breast cancer risk (i.e., positive personal or family history). In this study of 611 cases, the sensitivity increased to 90.5% when using CEM (for FFDM sensitivity was 52.4%), but specificity dropped from 90.5% (FFDM) to 76.1% (CEM) [15]. These preliminary studies confirm that CEM has a potential as a screening tool in women with high or intermediate breast cancer risk, or even as supplemental imaging tool in women with dense breasts.

However, an important disadvantage of performing CEM as screening tool is its increased radiation dose. Jeukens et al showed on a single commercially available unit that the radiation dose increased with 81% when CEM was used instead of full-field digital mammography (mean radiation dose of mammography being 1.55 mGy per exposure, compared with 2.80 mGy per CEM exposure) [16]. Hence, a complete FFDM (i.e., two breasts, two views) would result in an annual dose of 6.2 mGy. Considering the lifetime-attributable risk numbers and a 30-year screening period (according to our current national guidelines), the lifetime risk of radiation-induced breast cancer incidence is estimated to be 0.23% and its mortality 0.06% [25]. If CEM would be used as a screening tool, the annual dose would become 11.2 mGy, resulting in a lifetime risk of breast cancer incidence and mortality of 0.41% and 0.1%, respectively, during the 30-year screening period.

In theory, the use of single-view CEM could provide an interesting alternative, especially if sensitivity would not decrease significantly. The ideal study design to test this hypothesis would be a randomized controlled clinical trial, dividing women in a CEM- and MRI group for screening. However, the breast cancer incidence in this population is very low, requiring a larger number of study participants and a sufficiently long follow-up period to draw any final conclusions. Therefore, we opted to perform a retrospective study designed as a non-inferiority study first to evaluate whether the use of 1 V did not result in an unacceptable worse sensitivity for detecting breast cancer in our study population, while maintaining similar specificity when compared with 2 V.

With respect to the consensus evaluation, the lower limit of the 90% CI around the difference in sensitivity between 1 V and 2 V evaluation is − 15% and lies below the non-inferiority margin of − 10%. However, the entire 90% CI ranging from − 15 to − 5.3% does not exclude the non-inferiority margin. Therefore, we cannot conclude that 1 V is non-inferior to 2 V with respect to sensitivity, but neither can it be concluded that 1 V evaluations are inferior to 2 V evaluations (at a predefined non-inferiority margin of − 10%). With this chosen non-inferiority margin, formally the results are inconclusive with respect to non-inferiority [26]. However, based on the substantial and statistically significant decrease in sensitivity and overall diagnostic performance, we would not recommend 1 V CEM as an alternative reading strategy.

The most important causes for FN findings, and thus a decrease in sensitivity, were ILC and DCIS. Due to some selection bias caused by the study design, the prevalence of both ILC and DCIS in malignant cases was higher than would be expected (23.8% and 33.3%, respectively). Hypothetically, the sensitivity of both strategies would improve if these entities were less frequently observed in this population. Nevertheless, ILC shows no to subtle enhancement on 2 V CEM exams [27], while Houben et al recently showed that the use of 2 V CEM does not significantly increase its diagnostic performance for suspicious breast calcifications [28]. In summary, these diagnoses remain challenging even in 2 V CEM, and it is not plausible that the detection of these lesions will be better on 1 V CEM.

Another important disadvantage of CEM is the use of iodinated contrast agents, which can result in hypersensitivity reactions (or even anaphylactic shock) or can cause contrast-induced nephropathy. In a recent study of 839 patients, the incidence of mild or moderate hypersensitivity reactions during a CEM was 0.6%, without any severe reactions resulting in hospital admission or worse [29]. Although contrast-induced nephropathy might occur as a result of the administration of iodinated contrast agents, the incidence was recently estimated to be 2.6–2.7% in high-risk patients (i.e., with a glomerular filtration rate of 30–50 ml/min/1.73 m^2^) [30]. The expected incidence in our current population is expected to be lower, as it is not a high-risk group [29].

Considering these facts that CEM requires iodinated contrast agents and must be performed in 2 V, we agree with Jochelson et al that at present, CEM might be considered an alternative to breast MRI for screening high-risk patients, not a replacement. We support their proposal to perform larger prospective trials on this topic, but our results show that the CEM exam used in these studies should consist of a standard, two-view CEM exams of both breasts.

### Study limitations

Our study had several limitations. First, the cohort consisted of women undergoing CEM for an abnormality already suspected using a different modality, introducing some selection bias. However, the readers were blinded for the CEM indication and final diagnosis when reading the exams. Second, we used a blinded double-reading strategy for the analyses of this study, as it is similar to our national screening program. Other reading strategies, such as unblinded double reading, might have resulted in different observations. Another potential reading strategy, using a single radiologist aided by computer-aided detection (CAD) systems, was not feasible, since there are currently no approved CAD systems available for CEM. Third, the population that we used is not a high-risk population that would be considered for intensified screening (i.e., lifetime risk > 20%). These are more often young(er) women, with more often dense breasts, who can additionally express different breast cancer subtypes [31, 32]. CEM might be a suitable alternative screening method, since Lord et al showed a sensitivity of mammography and breast MRI combined of 94%, with a specificity varying between 77 and 96% [33]. Although these findings are in line with our observed diagnostic performance of 2 V CEM exams, it remains unclear how the results of our study population (i.e., a non-screening population not of high risk) would be applicable to the target population of (screening) high-risk patients. The use of CEM for screening high-risk patients needs to be studied further, but then using 2 V CEM exams (not 1 V). Finally, the follow-up period for benign diagnoses should preferably be more than 2 years for all lesions studied, while our current *mean* follow-up period is 21 months. Nevertheless, in a study using a similar population, Lalji et al showed that the chance of having overlooked a breast cancer when CEM was deemed “negative” is negligible [17].

## Conclusion

Non-inferiority of 1 V evaluations as alternative reading strategy to standard 2 V evaluations could not be concluded. 1 V evaluations had lower diagnostic performance compared with 2 V evaluations.

## Abbreviations

1 V: Single-view contrast-enhanced mammography
2 V: Double-view contrast-enhanced mammography
AUC: Area under ROC curve
CEDM: Contrast-enhanced dual-energy mammography
CEM: Contrast-enhanced mammography
CESM: Contrast-enhanced spectral mammography
CI: Confidence interval
DCIS: Ductal carcinoma in situ
FFDM: Full-field digital mammography
FN: False negative
FP: False positive
ILC: Invasive lobular carcinoma
MRI: Magnetic resonance imaging
NPV: Negative predictive value
NST: No special type
PPV: Positive predictive value
ROC: Receiver operating characteristic
TN: True negative
TP: True positive
